# The Aldose Reductase Inhibitor Epalrestat Maintains Blood–Brain Barrier Integrity by Enhancing Endothelial Cell Function during Cerebral Ischemia

**DOI:** 10.1007/s12035-023-03304-z

**Published:** 2023-03-20

**Authors:** Tongshuai Zhang, Jinrong Wu, Xinmin Yao, Yao Zhang, Yue Wang, Yang Han, Yun Wu, Zhenyu Xu, Jing Lan, Siyu Han, Haifeng Zou, Qixu Sun, Dandan Wang, Jingyu Zhang, Guangyou Wang

**Affiliations:** 1 Department of Neurobiology, Heilongjiang Provincial Key Laboratory of Neurobiology, Harbin Medical University, Harbin, 150081 Heilongjiang China; 2 Department of Anaesthesiology, The First Affiliated Hospital of Harbin Medical University, Harbin, 150001 Heilongjiang China; 3 Traditional Chinese Medicine, Heilongjiang University of Chinese Medicine, Harbin, 150040 Heilongjiang China; 4 Department of Anesthesiology, Second Affiliated Hospital of Air Force Medical University, Xi’an, 710032 Shaanxi China; 5 The Medical Department of Neurology, The Second Affiliated Hospital of Harbin Medical University, Harbin, 150001 Heilongjiang China; 6 Department of Gastroenterology, Penglai People’s Hospital, Yantai, 264117 Shandong China; 7 Wu Lian De Memorial Hospital, The First Affiliated Hospital of Harbin Medical University, Harbin, 150001 Heilongjiang China; 8 The Medical Department of Neurology, The Fourth Affiliated Hospital of Harbin Medical University, Harbin, 150001 Heilongjiang China

**Keywords:** Cerebral ischemia, Blood brain barrier (BBB), Brain microvascular endothelial cells (BMVECs), Aldose reductase (AR), Epalrestat, Autophage

## Abstract

Excessive activation of aldose reductase (AR) in the brain is a risk factor for aggravating cerebral ischemia injury. Epalrestat is the only AR inhibitor with proven safety and efficacy, which is used in the clinical treatment of diabetic neuropathy. However, the molecular mechanisms underlying the neuroprotection of epalrestat remain unknown in the ischemic brain. Recent studies have found that blood–brain barrier (BBB) damage was mainly caused by increased apoptosis and autophagy of brain microvascular endothelial cells (BMVECs) and decreased expression of tight junction proteins. Thus, we hypothesized that the protective effect of epalrestat is mainly related to regulating the survival of BMVECs and tight junction protein levels after cerebral ischemia. To test this hypothesis, a mouse model of cerebral ischemia was established by permanent middle cerebral artery ligation (pMCAL), and the mice were treated with epalrestat or saline as a control. Epalrestat reduced the ischemic volume, enhanced BBB function, and improved the neurobehavior after cerebral ischemia. *In vitro* studies revealed that epalrestat increased the expression of tight junction proteins, and reduced the levels of cleaved-caspase3 and LC3 proteins in mouse BMVECs (bEnd.3 cells) exposed to oxygen–glucose deprivation (OGD). In addition, bicalutamide (an AKT inhibitor) and rapamycin (an mTOR inhibitor) increased the epalrestat-induced reduction in apoptosis and autophagy related protein levels in bEnd.3 cells with OGD treatment. Our findings suggest that epalrestat improves BBB function, which may be accomplished by reducing AR activation, promoting tight junction proteins expression, and upregulating AKT/mTOR signaling pathway to inhibit apoptosis and autophagy in BMVECs.

**Supplementary Information:**

The online version contains supplementary material available at 10.1007/s12035-023-03304-z.

## Introduction

Stroke is a serious neurological disease with high incidence, disability, and mortality rates worldwide. More than 80% of strokes are initially ischemic, which causes vascular thrombosis due to atherosclerosis [1]. Cerebral ischemia leads to hypoxia, oxidative stress, and inflammatory responses, which are important causes of acute necrosis, apoptosis, and autophagy in brain cells [2, 3]. The blood–brain barrier (BBB) is an important brain structure that protects the brain from harmful peripheral substances. The integrity of the BBB is mainly determined by brain microvascular endothelial cells (BMVECs), astrocytes, pericytes, and the basement membrane. Under physiological conditions, BMVECs maintain the stability of the internal environment of the neurovascular system through paracrine, endocrine, and autocrine functions [4]. Recent studies have found that BBB damage after cerebral ischemia is mainly caused by the extensive necrosis and apoptosis of BMVECs, as well as the destruction of tight junction [5, 6]. In addition, neutrophils and macrophages are attracted to the ischemic area, releasing pro-inflammatory factors, reactive oxygen species, further damaging endothelial cells, aggravate BBB damage [7]. Therefore, finding a strategy to improve the survival of BMVECs after cerebral ischemia, and thus maintain the integrity of the BBB, has become a key goal for the treatment or prevention of cerebral ischemia injury.

The production of reactive oxygen species (ROS) is under tight control in healthy cells; however, overproduction of ROS during cerebral ischemia causes oxidative stress injury, which leads to acute necrosis, apoptosis, and autophagy in the brain cells, accompanied by serious damage to the neural network system and consequent BBB disruption [8]. ROS formed after cerebral ischemia can activate the polyol pathway enzyme aldose reductase (AR; also known as AKR1B1) by modifying its cysteine residues to sulfenic acids [9]. Excess activation of AR in the mouse brain was shown to deplete intracellular NADPH, thereby reducing glutathione levels and further mediating ROS-induced signals that contribute to the inflammatory response in cerebral ischemia [10]. Similarly, overexpression of human AR in macrophages was reported to promote inflammation and accelerate atherosclerotic progression in diabetic mice [11]. Recent studies have shown that up-regulation of AR expression in hippocampal neurons can promote the production of NLRP3 inflammatory corpuscles and damage the remodeling of vascular neurons after a remote ischemic injury in diabetes [12, 13]. In central nervous system (CNS) diseases, especially spinal cord injury and ischemic stroke, the increase in AR levels in activated microglia promotes their polarization to the M1 macrophage type, which in turn aggravates the inflammatory reaction and injury degree [14, 15]. Although studies have shown that an increase in AR levels in damaged endothelial cells causes cell death via necrotic or apoptotic mechanisms [16, 17], the role of AR accumulation in cerebral ischemia-induced BMVEC dysfunction and cell viability remains unknown.

As a reductase, activated AR reduces glucose to sorbitol, which cannot readily pass through the cell membrane. Sorbitol accumulation causes changes in the cell’s osmotic pressure and oxidative stress, leading to tissue dysfunction, intracellular signal changes, and extensive cell death [18, 19]. Therefore, inhibiting AR overproduction is a key target to maintain the normal metabolism of injured cells. Recent studies have confirmed that the use of AR inhibitors can effectively reverse the adverse consequences of cells induced by excessive AR production [20]. Despite the development of several AR inhibitors, to date, they have mostly been applied in preclinical studies or clinical trials. Currently, epalrestat is the only commercial AR inhibitor available for clinical application in a few Asian countries, which has been proven safety and efficacy without major side effects, and able to crosses the blood–brain barrier [21–25]. Previous studies have focused on the effect of epalrestat to reduce secondary diabetic complications, including retinopathy, neuropathy, and atherosclerosis, caused by the overexpression of AR [26]. However, further research is revealing the broader roles and potential of epalrestat in the protection of cancer and CNS diseases.

Epalrestat was shown to significantly suppress cancer stem cell properties, tumorigenicity, and metastasis of basal-like breast cancer cells [27, 28]. Our previous study confirmed that epalrestat can effectively reverse the activation of microglia induced by a high-salt diet and reduce the inflammatory response after cerebral ischemia in mice [29]. However, the underlying mechanism and role of epalrestat in protection of the BBB after cerebral ischemia remain unclear, especially in promoting the maintenance of the barrier function of BMVECs.

Previous studies have found that antioxidants, anti-inflammatory factors, and cell signaling pathway regulators can effectively improve endothelial cell dysfunction [30–33]. As the central regulator of multiple cellular signaling pathways, mammalian rapamycin target protein (mTOR) maintains cell metabolic processes such as cell growth, apoptosis, and autophagy [34, 35]. As upstream cellular signal targets of mTOR, adenosine monophosphate activated protein kinase (AMPK) and class I phosphoinositide-3 kinase (PI3K) can positively regulate mTOR activation [36]. Recent studies have found that under conditions of glucose deprivation, AMPK is activated, mTOR is inhibited, and cardiomyocyte autophagy is promoted [37, 38]. Protein kinase B (AKT), a downstream target of the PI3K signaling pathway, regulates cell proliferation, differentiation, and apoptosis, and the PI3K/AKT/mTOR pathway is involved in the regulation of autophagy in neural stem cells [39]. However, few studies have reported the regulatory effect of epalrestat on the mTOR signaling pathway. One study showed that epalrestat improved the effects of sorafenib on liver cancer by regulation of Bcl-2/caspase-3-mediated apoptosis and inhibiting the mTOR pathway to activate autophagy [40]. Epalrestat was also found to regulate the mTOR pathway to inhibit apoptosis and autophagy in oral cancer cells [41]. Therefore, we hypothesized that epalrestat could inhibit the apoptosis and autophagy of BMVECs through the ATK/mTOR signaling pathway to maintain endothelial cell barrier function, and thus protect against BBB damage and further injury in cerebral ischemia.

To evaluate this hypothesis, we examined the protective effect of epalrestat on BMVEC dysfunction induced by cerebral ischemia in a mouse model. We further investigated the effect of epalrestat on excessive AR production, tight junction proteins expression and induction of apoptosis and excessive autophagy after ischemia. *In vitro* experiments with a mouse BMVEC cell line under oxygen–glucose deprivation (OGD) conditions were used to examine the underlying mechanism via the effects of epalrestat treatment on the AR/AKT/mTOR signaling pathway activated by ROS. Overall, these results can highlight epalrestat as a new clinical drug for the treatment of cerebral ischemia.

## Materials and Methods

### Animals, Model Establishment, and Treatment

C57/BL6 male mice (18–22 g; Liaoning Changsheng Biotechnology Ltd., Liaoning, People’s Republic of China) were used in this experiment. The mice received normal chow and tap water, and were raised in accordance with the guidelines of the Care and Use of Laboratory Animals published by the China National Institute of Health and the ARRIVE guidelines. All experiments were approved by the Ethics Committee of the Harbin Medical University.

The mice were observed and weighed daily, and the model was established when the weight reached 20–25 g. Cerebral ischemia was induced by permanent middle cerebral artery ligation (pMCAL) as previously described [42]. The mice were first anesthetized with 2% nembutal (5 mL/kg). An incision was made from the left temporal muscle with a spring scissor, and then bone rongeurs were used to remove a piece of the skull to slightly expose the middle cerebral artery. Finally, the distal part of the artery was ligated with a vessel cauterizer to establish the cerebral ischemia model. In the sham-operated group, the vessels were bluntly dissected but without ligation.

After modeling, the sham-operated and cerebral ischemia model mice were divided into two groups: one group was administered epalrestat by gavage twice a day at 50 mg/kg (SML0527, Sigma Chemical Company, St. Louis, Missouri), which dissolved in control saline, and the other was administered equal amounts of saline (NaCl) as the control group. In addition, in order to judge the effect of epalrestat dose on the experimental results, we set three concentrations of 25 mg/kg, 50 mg/kg and 100 mg/kg for experimental verification.

### Cranial Ischemia Model Confirmation

The mice were euthanized with nembutal anesthesia 12 h, 1 d, 3 d, and 5 d after model establishment. After intracardiac perfusion with phosphate-buffered saline (PBS), the mouse brain tissue was carefully removed. Brain slices (2 mm) were cut along the coronal suture with a brain slicer and incubated in 2% 2,3,5-triphenyltetrazolium chloride (TTC) solution (in PBS) for 30 min at room temperature. Areas without red staining by the TTC reagent were assumed to be injured. All sections were photographed with a digital camera and the infarct areas (white) were measured blindly using Image J software.

### Assessment of BBB Damage

Evans blue is widely used to assess the integrity of the BBB. After modeling, the mice were intravenously injected with 4% Evans blue dye (E2129, Sigma-Aldrich) at 100 μL/20 g via the tail vein, and the body quickly turned blue. The vital signs were carefully monitored for 30 min, and then the mice were sacrificed and transcardially perfused with cold PBS to flush away the blood and Evans Blue from the blood vessels. The ischemic lateral brain tissue was quickly removed and placed in a solution of 50% trichloroacetic acid in PBS (1.5 mL). The brains were homogenized, sonicated in trichloroacetic acid, and centrifuged at 12,000 rpm for 20 min. The supernatant was collected, and the fluorescence emission was measured at 680 nm (with an excitation wavelength of 620 nm) using an ultraviolet spectrophotometer.

### Neurological Score

After model establishment, the mice were placed in a cage alone to observe their overall state and behavior. The neurological score [43] was calculated as follows: 0 points, normal behavior; 1 point, cannot fully extend the right front legs; 2 points, turning around in a circle; 3 points, falling onto the right side; 4 points, cannot move on own and loss of consciousness; 5 points, death.

The effects of cerebral ischemia and epalrestat treatment on sensorimotor abilities were evaluated based on forelimb use, since animals with unilateral ischemic brain injury show forelimb preference. The forelimb movements of each mouse were analyzed in a transparent plexiglass rearing cylinder 9 cm in diameter and 15 cm in height. The size of the cylinder is sufficient to allow for free movement, and its weight prevents it from moving during support [44]. The mice were trained three times a day for approximately 5 min each for the 3 d prior to the trial. After modeling, each mouse was individually placed in the cylinder and observed for 5 min. To evaluate the sensorimotor deficit, the percentage of weight-bearing episodes (braces) on the side of the cylinder that were initiated with the non-impaired (ipsilateral), impaired (contralateral), and both forepaws were calculated in each animal after pMCAL. The initial forepaw placement of each weight-bearing contact with the wall was recorded as right or left. In this experiment, the damage was induced on the left side, and therefore we compared the times of using the right paw to the total number of times of weight-bearing contact with a forelimb.

### Immunofluorescence and TUNEL Staining

The brains were collected from the mice in the different groups and fixed to obtain 10-μm-thick cryosections. The cryosections were washed with 0.01 mol/L PBS (pH = 7.4) and blocked with 1% bovine serum albumin (BSA) for 1 h. The cryosections were then incubated with anti-CD31 (ab7388, Abcam, 1:500), anti-cleaved-caspase3 (ab214430, Abcam, 1:500), anti-occludin (ab216327, Abcam, 1:500), anti-F4/80 (30325, Cell Signaling Technology, 1:800), anti-LC3B (L7543, Sigma-Aldrich, 1:500), anti-P62 (ab109012, Abcam, 1:500), anti-LAMP1 (ab225762, Abcam, 1:200), anti-GFAP (ab53554, Abcam, 1:500), anti-AR (sc-166918, Santa Cruz, 1:200), and anti-ZO-1 (ZO1-1A12, Thermo Scientific, 1:200) antibodies overnight at 4 °C. The sections were incubated with secondary antibodies, including fluorescein isothiocyanate (FITC)-conjugated donkey-anti-goat/rat IgG and TRITC-conjugated donkey anti-rabbit IgG (Jackson, 1:500) at room temperature for 1 h. The sections were washed with flowing water three times for 5 min and incubated for 5 min with 4′,6-diamidino-2-phenylindole dihydrochloride (DAPI; 28718–90-3, Sigma-Aldrich) to stain the nuclei. Apoptotic cells were detected using an *in situ* cell death (TUNEL) detection kit (Roche). After staining with DAPI, the samples were treated with 0.3% H_2_O_2_ for 10 min to remove endogenous peroxides at room temperature and then rinsed four times with 0.01 mol/L PBS (5 min each time). The sections were then incubated with the TUNEL reaction mixture (solution A/B, 1:9) in a humidified chamber at 37 °C for 1 h under dark conditions. The sections were washed three times with 0.01 mol/L PBS and placed in a dark chamber at 37 °C for 30 min. Subsequently, the specimens were washed four times with 0.01 mol/L PBS (5 min each time). Finally, the sections were mounted and observed under a confocal microscope (Zeiss, Germany). A negative control was established for each group to which no primary antibody was added. The rest of the steps were consistent; however, no positive fluorescent areas were observed.

### Flow Cytometry

Brain hemispheres were collected from the mice in different groups after intracranial perfusion with D-Hanks’ balanced salt solution after modeling for flow cytometric analysis. A 70–30% Percoll gradient centrifugation protocol was used for flow cytometry analysis of neutrophils marked with CD45-Percp (557235, BD Biosciences, 1:100), CD11b-APC (553312, BD Biosciences, 1:100), and Ly6G-FITC (551460, BD Biosciences, 1:100) infiltrating the brain with a FACS Calibur flow cytometer (BD Biosciences). All antibodies and isotype-matched controls were purchased from BD Biosciences (San Diego, CA, USA), and the analysis was conducted using FlowJo software.

The flow cytometry procedure for detecting macrophages and neutrophils in peripheral blood circulation was different from that described above. Blood was collected from the eyeballs of the mice after modeling, and the blood was placed in an anticoagulant tube. After adding red blood cell lysate, the blood was gently vortexed and shaken. Then the cell mixture was filtered, and the cells were transferred to a 96-well plate. After breaking the membrane, antibodies were added for incubation. Related flow cytometric antibodies were: CD45-Percp (557235, BD Biosciences, 1:100), CD11b-APC (553312, BD Biosciences, 1:100), and Ly6G-PE (127608, Biolegend, 1:100)、F4/80-FITC (123107, Biolegend, 1:100), Fixable Viability Dye eFluor™ 780 (65–0865-18, eBiosciences, 1:2000).

### Cell culture and Treatment

Mouse brain-derived endothelial cells (bEnd.3) were obtained from the American Type Culture Collection (Manassas, VA, USA). The cells were cultured in Dulbecco’s modified Eagle medium (DMEM) supplemented with 10% fetal bovine serum (FBS), penicillin (100 U/mL), and streptomycin (100 mg/mL), and maintained at 37 °C in a 5% CO_2_ incubator. When the cells state was better, epalrestat was added at 30 μm/L, untreated cells served as the control group. Additionally, bicalutamide (an AKT inhibitor) and rapamycin (an mTOR inhibitor) were added to the culture medium along with epalrestat to analyze the effect of epalrestat on the AR/AKT/mTOR signaling pathway. The cells were passaged about ten times.

### *In Vitro* Ischemia Model

OGD is widely used as an *in vitro* model of ischemic stroke. The cells were placed in a modular incubator after the medium was replaced with glucose-free DMEM. The chamber was then filled with a mixture of 95% N_2_ and 5% CO_2_, and the temperature of the chamber was set to 37 °C. The cells were exposed to the OGD condition for 2 h, 4 h, 6 h, and 8 h.

### Cell Viability Assay

To test the effects of epalrestat on endothelial cell activity, the bEnd.3 cell viability was determined using a Cell Counting Kit-8 (CCK-8) assay (Dojindo, Japan). The cells(1 × 10^4^/well) were seeded in 96-well culture plates and incubated overnight in DMEM containing 10% FBS. Epalrestat was added into cells at 0, 10, 30, 50 μmol/L, followed by exposure to OGD after 24 h. The CCK-8 solution (10 μL) was added to each well of the plate and the plates were incubated for 4 h. The absorbance at 450 nm was measured using an ultraviolet spectrophotometer. Furthermore, to test the effects of bicalutamide and rapamycin on endothelial cell activity, bicalutamide was added to cells at 0,0.5,1.0,1.5 μmol/L, and rapamycin at 0, 0.1, 0.3, 0.5, 1.0 μmol/L. The rest of the steps were the same as above, and the results was in Supplementary Materials.

### Permeability Assays

Permeability assays were conducted as previously described [45]. The bEnd.3 cells (1 × 10^4^) were plated on top of a 3-μm pore-size upper Transwell chamber (Corning, USA) with epalrestat for 1 day and then the medium was replaced with glucose-free DMEM containing 50 μg/mL of BSA-FITC (A9771, Sigma) to the Transwell upper chambers with exposure to the OGD condition for 4 h. Media (both 100 μL) were collected from the upper and lower chambers of each well, and the fluorescence intensity was measured using an FL600 microplate fluorescent reader (Biotek). Cells not exposed to OGD were cultured in a cell incubator (5% CO_2_) in glucose-free DMEM as a control group for comparison.

### Protein Preparation and Western Blot

Protein samples were extracted from the cultured bEnd.3 cells *in vitro* and from the ischemic brains of the mice. RIPA lysis buffer (Santa Cruz Biotechnology, Santa Cruz, CA, USA) was added to the cell/tissue homogenate, and the lysed proteins were centrifuged at 12,000 × *g* for 15 min at 4 °C. The supernatants were collected for protein concentration measurements using a BCA protein assay kit (Pierce, Rockford, IL, USA). The protein samples were loaded on 10% Tris–HCl sodium dodecyl sulfate–polyacrylamide gels (Bio-Rad Laboratories, Hercules, CA, USA) for electrophoresis (120 V, 60 min) and then transferred onto a polyvinylidene fluoride membrane for blocking (block solution, 5% non-fat milk solution dissolved in Tris-buffered saline with Tween). The membranes were incubated with the following primary antibodies overnight at 4 °C: anti-β-actin (TA-09, ZSGB-BIO, 1:1000), anti-AR (sc-166918, Santa Cruz Biotechnology, 1:500), anti-occludin (ab216327, Abcam, 1:1000), anti-cleaved-caspase3 (ab214430, Abcam, 1:1000), anti-Bcl2 (sc-783, ZSGB-BIO, 1:1000), anti-LC3B (L7543, Sigma-Aldrich, 1:1000), anti-Beclin1 (ab207612, Abcam, 1:1000), anti-ZO-1 (ZO1-1A12, Thermo Scientific, 1:1000), anti-Bax (sc-7480, Proteintech, 1:1000), anti-AKT (ab38449, Abcam, 1:1000), anti-p-AKT (4060, Cell Signaling Technology, 1:1000), anti-mTOR (ab109268, Abcam, 1:1000), and anti-p-mTOR (5536, Cell Signaling Technology, 1:1000). The membranes were then incubated with goat anti-rabbit or anti-mouse secondary antibodies (Santa Cruz Biotechnology). The protein levels were normalized to the level of β-actin, and immunoreactivity signals were detected relative to the corresponding control.

### Cell Apoptosis Assay

Cell apoptosis was analyzed using the Annexin V-FITC/PI Apoptosis Detection Kit (Vazyme Biotech Co., Ltd., Nanjing, China) with a FACS Calibur flow cytometer (BD). When the cells reached the wall of the bottle, epalrestat was added according to the experimental conditions, followed by OGD treatment. After OGD, the cells were counted and seeded in 96-well plates at a density of 1 × 10^6^ cells per well. The supernatants were then centrifuged, discarded, and the antibodies were configured according to kit standards using buffer buffer, 50 μl of antibodies were added to each well, the cells were resuspended, and incubated at room temperature for 20 min, finally, buffer was added to stop antibody incubation.

### Statistical Analysis

All quantitative data are expressed as the mean ± standard deviation. Statistical analysis was performed using GraphPad Prism software (version 6.0). Unpaired Student’s t-tests were used to compare data between two groups. One-way or two-way analysis of variance with a Tukey post-hoc test was used for comparisons among three or more groups. Differences were considered statistically significant at P < 0.05.

## Results

### Epalrestat Reduced Cerebral Ischemia Injury and Preserved BBB Integrity *In Vivo*

**Fig. 1:**
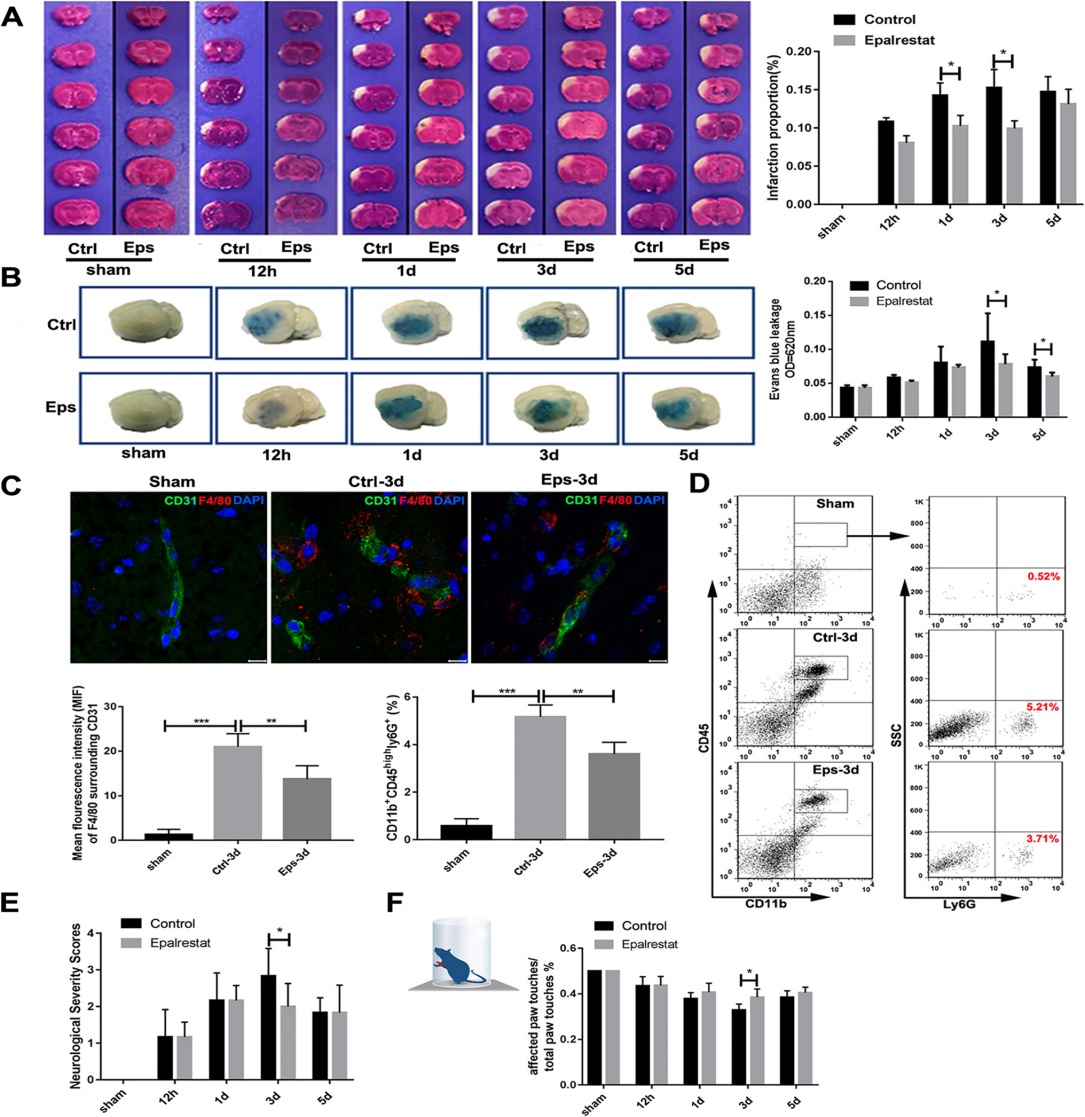
Epalrestat reduced cerebral ischemia injury and preserved BBB integrity in vivo. (**A**) Representative TTC-stained brain sections from mice in different groups after pMCAL. The infarct lesions remain unstained, and normal brain tissue is stained red (* *P* < 0.05, ** *P* < 0.01, *n* = 8 per group). (**B**) Representative images of Evans blue-stained brain tissue. Epalrestat reduced Evans blue leakage after pMCAO at 3 d and 5 d. (* *P* < 0.05, *n* = 8 per group). (**C**) Immunofluorescence double staining showed BMVECs and macrophages around the peri-infarction area. F4/80 is marked red, and CD31 is marked green. Scale bar = 10 μm. (** *P* < 0.01, *** *P* < 0.001, *n* = 5 per group). (**D**) Neutrophil was identified as CD11b^+^CD45^high^ Ly6G^+^cells. (** *P* < 0.01, *** *P* < 0.001, *n* = 5 per group). (**E**) Neurological scores in different groups after pMCAL. (* *P* < 0.05, *n* = 8 per group). (**F**) The changes of sensorimotor disorders in different groups of mice after pMCAL. (* *P* < 0.05, *n* = 8 per group). The values represent the mean ± SD

To investigate the effect of epalrestat on the cerebral ischemia-induced infarct volume and BBB permeability, mice were administered epalrestat and control saline from 1 h after pMCAL to the final day. The pMCAL group exhibited a significant increase in infarct volume compared with that of the sham group. However, epalrestat significantly reduced the infarct volume compared with that of the control (saline) group at 1 d and 3 d after pMCAL (Fig. 1A). The amount of Evans blue dye extravasation in the ischemic cerebral hemisphere increased 1 d after pMCAL compared with that of the two sham groups. However, epalrestat treatment significantly attenuated the extravasation of Evans blue dye compared with that of the control group after pMCAL (Fig. 1B). In order to verify whether epalrestat protects against cerebral ischemia injury in a dose dependent manner, we used different concentrations of epalrestat to compare the effects on ischemic brain volume and barrier function of BBB. Statistical analysis showed that 25 mg/kg epalrestat had no significant effect on cerebral ischemic injury, while 100 mg/kg epalrestat and 50 mg/kg epalrestat had similar effects on the reduction of ischemic brain volume and the maintenance of blood brain barrier function. Therefore, 50 mg/kg of epalrestat was the optimal concentration for this study (Fig. S1, S2).

Because a complete BBB structure effectively prevents the infiltration of inflammatory cells, we observed the number of macrophages and neutrophils in the ischemic hemisphere among the groups. The immunofluorescence results showed that compared with the sham group, the number of macrophages infiltrating the ischemic core area in the ischemic hemisphere was significantly increased at 3 days after pMCAL. However, the number of macrophages around endothelial cells decreased in the epalrestat group compared with that of the control group (Fig. 1C). Flow cytometry further showed that the percentage of neutrophils (CD11b^+^CD45^high^Ly6G^+^) infiltrated in ischemic hemisphere of epalrestat group was lower than that of the control group after pMCAL (Fig. 1D). The results of peripheral blood flow cytometry in different groups of mice showed that the percentage of macrophages (CD45^+^CD11b^+^F4/80^+^) and neutrophils (CD45^+^CD11b^+^Ly6G^+^) in peripheral blood circulation increased significantly after 3 days of cerebral ischemia compared with Control + Sham and Eps + Sham groups; Statistical analysis showed that there was no significant difference in the percentage of macrophages and neutrophils between Control + 3d and Eps + 3d groups (Fig. S3).

The neurobehavioral scores of the pMCAL groups were significantly higher than those of the two sham groups. Moreover, the neurobehavioral score of the epalrestat group was lower than that of the control group 3 d after pMCAL (Fig. 1E). In the sensorimotor bias chamber test, the mice in the sham groups primarily showed symmetrical use of their forepaws when bracing, with approximately 20% ipsilateral and contralateral paw use. Epalrestat treatment significantly improved this asymmetry compared with that of the control group 3 d after pMCAL (Fig. 1F).

Collectively, these results indicated that epalrestat contributes to a decrease in the volume of cerebral ischemia, maintains the structural integrity of the BBB, reduces the number of infiltrating inflammatory cells, and improves the neuromotor behavior of mice after cerebral ischemia.

### Epalrestat Attenuated the Degradation of Tight Junction (TJ) Proteins after Ischemia *In Vivo*

**Fig. 2:**
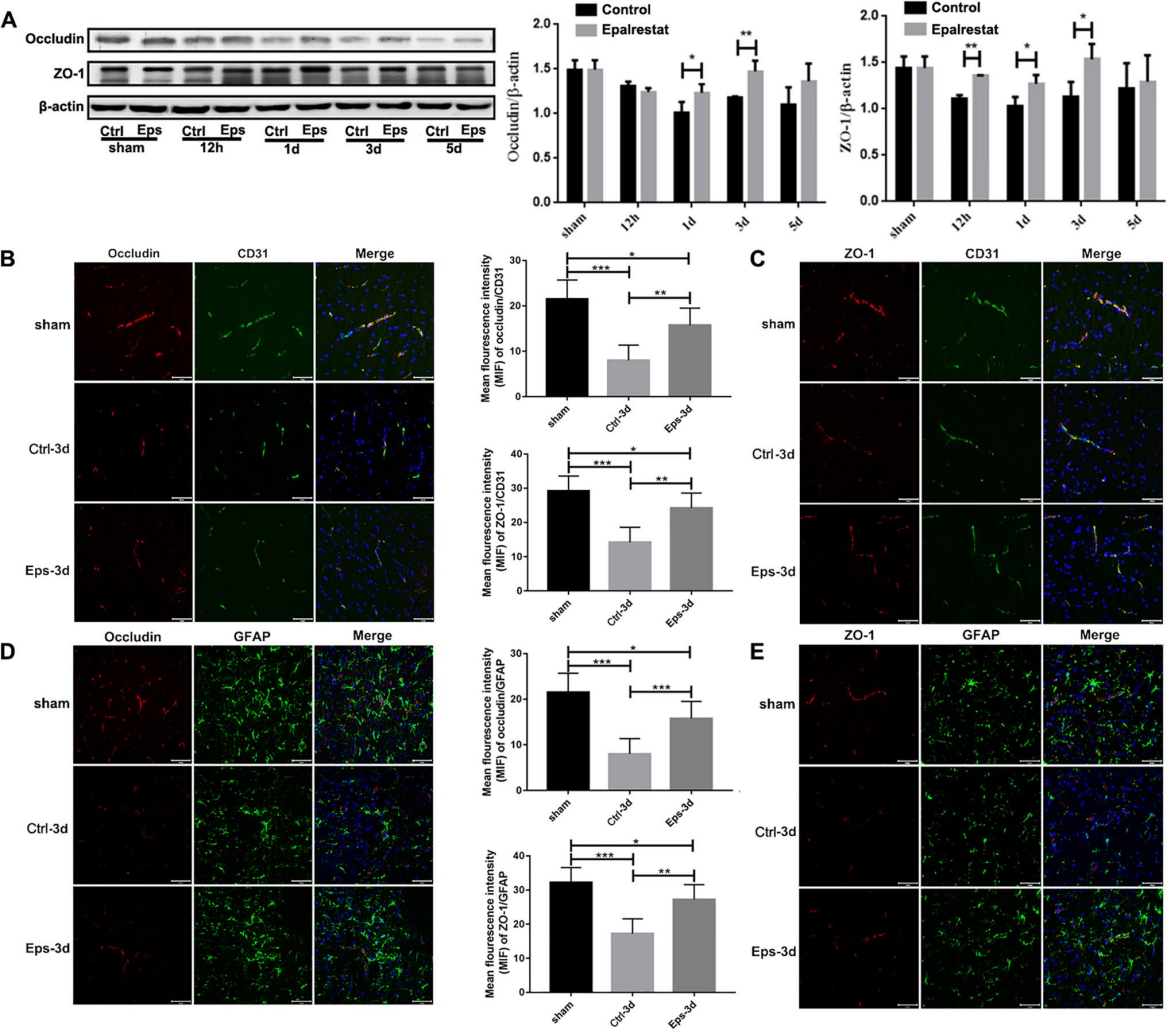
Epalrestat attenuated the degradation of tight junction (TJ) proteins after cerebral ischemia *in vivo*. (**A**) Expression of TJ proteins (ZO-1 and occludin) was reduced after permanent middle cerebral artery ligation (pMCAL), and the effect was reversed with epalrestat (Eps) treatment at 12 h, 1 d, and 3 d. (* *P* < 0.05, ** *P* < 0.01; *n* = 5 per group). (**B**, **C**) Representative immunofluorescence images of TJ proteins in endothelial cells after pMCAL. ZO-1 and occludin staining is marked red, and CD31 staining is marked green. Scale bar = 20 μm. (*P < 0.05, **P < 0.01, ***P < 0.001; *n* = 5 per group). (**D**, **E**) Immunofluorescence double staining of astrocytes and TJ proteins present around the peri-infarction area. ZO-1 and occludin are marked red, and GFAP is marked green. Scale bar = 20 μm. (*P < 0.05, **P < 0.01, *** *P* < 0.001;* n* = 5 per group). Data represent the mean ± SD

To further examine the role of epalrestat in maintaining BBB integrity, changes in TJ proteins (ZO-1 and occludin) in vascular endothelial cells following pMCAL were detected by Western blot and immunofluorescence staining. Western blotting results showed that the expression of TJ proteins in the brain of control group mice decreased significantly after pMCAL; epalrestat treatment reversed this loss of TJ protein expression 1 d and 3 d after pMCAL, but did not affect the expression of TJ proteins in the sham groups (Fig. 2A). Moreover, double immunofluorescence staining showed that the morphology and expression of TJ proteins in the ischemic hemisphere was disrupted and lost, respectively, in the control group after pMCAL, whereas epalrestat treatment prevented the degradation of TJ proteins and maintained BBB integrity (Fig. 2B-C). Because astrocyte end-feet and endothelial cell TJ proteins are essential for the maintenance of BBB homeostasis [46], we examined the structural integrity of the BBB in the ischemic penumbra area through double-staining of endothelial cells and astrocytes. The BBB structure was relatively complete in the sham group, and was more complete in the infarct area of the epalrestat group compared with that of the control group after pMCAL (Fig. 2D-E). These findings demonstrated that epalrestat reverses the loss of TJ proteins in BMVECs *in vivo* after cerebral ischemia injury.

### Epalrestat Effectively Reduced the AR Expression and Apoptosis of Endothelial Cells *In Vivo* after Ischemia

**Fig. 3:**
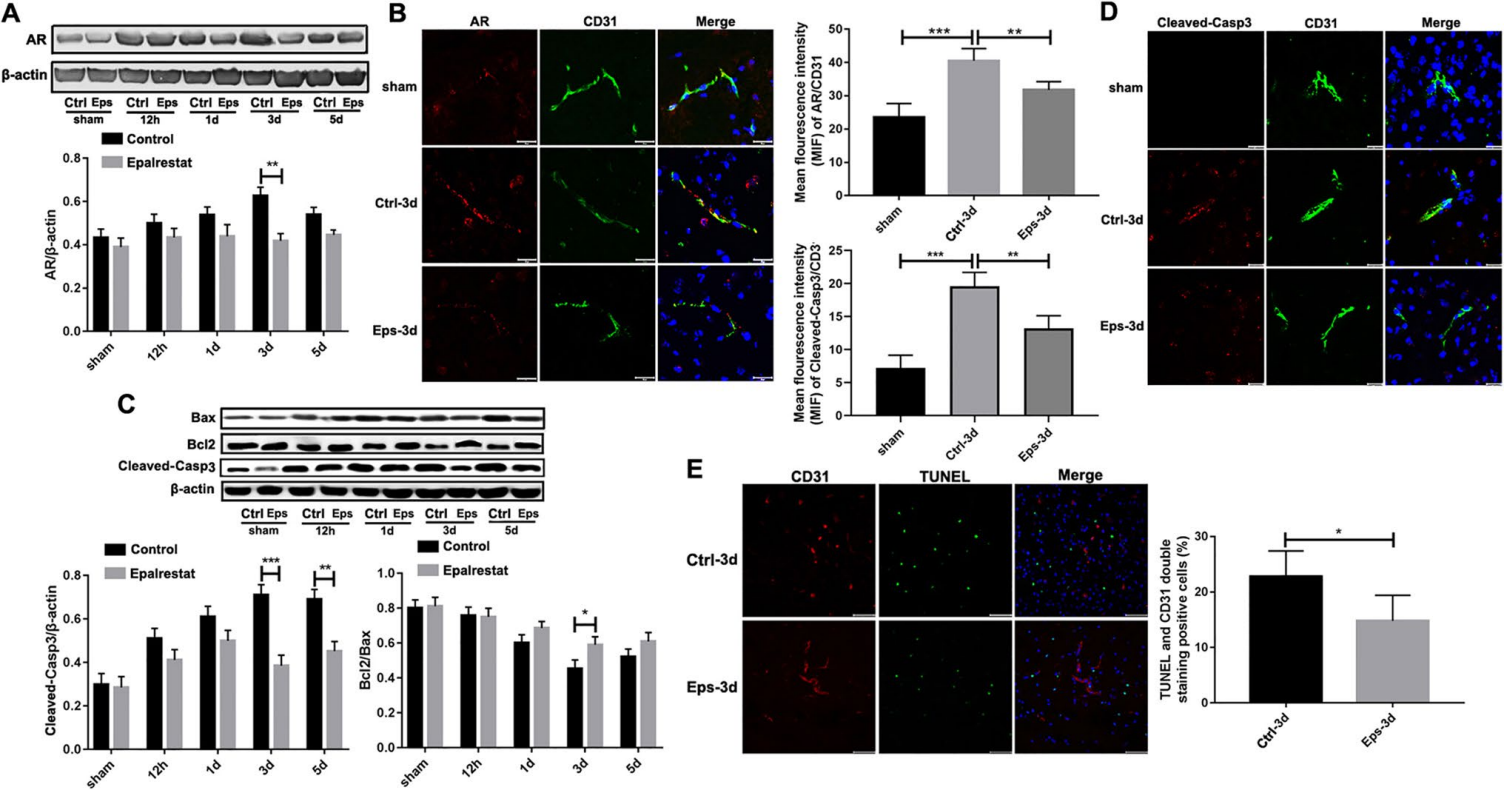
Epalrestat effectively reduced aldose reductase (AR) expression and the apoptosis of endothelial cells *in vivo* after ischemia. (**A**) AR protein expression in different groups after permanent middle carotid artery ligation (pMCAL). (**P < 0.01; *n* = 5 per group). Eps = epalrestat. (**B**) AR (red) and CD31 (green) co-staining around the peri-infarction area in different groups. Scale bar = 10 μm. (**P < 0.01, ***P < 0.001; *n* = 5 per group). (**C**) Expression of apoptosis-related proteins in the ischemic brains. (*P < 0.05, ** *P* < 0.01, ***P < 0.001; *n* = 5 per group). (**D**) Cleaved-caspase3 (red) and CD31 (green) co-staining around the peri-infarction area in different groups. Scale bar = 10 μm. (**P < 0.01, ***P < 0.001; *n* = 5 per group). (**E**) Cerebral microvascular endothelial cell apoptosis in the ischemic brain tissue as detected by TUNEL (green)/CD31 (red) immunofluorescence double staining. Scale bar = 10 μm. (*P < 0.05; *n* = 5 per group). Data represent the mean ± SD

The expression of AR proteins was greatly increased in ischemic brain tissues of the control group after pMCAL compared with that of the sham group, and epalrestat significantly reduced the expression of AR 3 d after pMCAL (Fig. 3A). Immunofluorescent staining for AR surface markers on endothelial cells in the ischemic penumbra of the ischemic hemisphere showed that the mean fluorescence intensity of CD31/AR in the epalrestat group was significantly lower than that in the control group at 3 d following modeling (Fig. 3B).

Western blotting showed that the expression of the apoptosis marker cleaved-caspase 3 in the ischemic brain tissue was significantly higher than that in the sham group. At 3 d and 5 d after pMCAL, the expression of cleaved-caspase 3 in the epalrestat group was significantly lower than that in the control group. Similarly, the ratio of Bcl-2/Bax proteins was significantly reduced in the control group compared with that of the two sham groups, and this effect was significantly reversed in the epalrestat group 3 d after pMCAL (Fig. 3C). Immunofluorescence staining of the apoptosis marker cleaved-caspase3 on the endothelial cell in the ischemic penumbra of the ischemic hemisphere showed that the mean fluorescence intensity of CD31/cleaved-caspase3 in the epalrestat group was significantly lower than that in the control group 3 d after modeling (Fig. 3D). Moreover, the number of TUNEL-positive endothelial cells was greatly increased in the control group, and epalrestat reversed this effect 3 days after ischemia (Fig. 3E). These results indicated that epalrestat could effectively reduce AR expression and apoptosis in endothelial cells after cerebral ischemia.

### Epalrestat Maintains BBB Function by Up-Regulating TJ Proteins and Inhibiting the Apoptosis of Epithelial Cells after OGD *In Vitro*

**Fig. 4:**
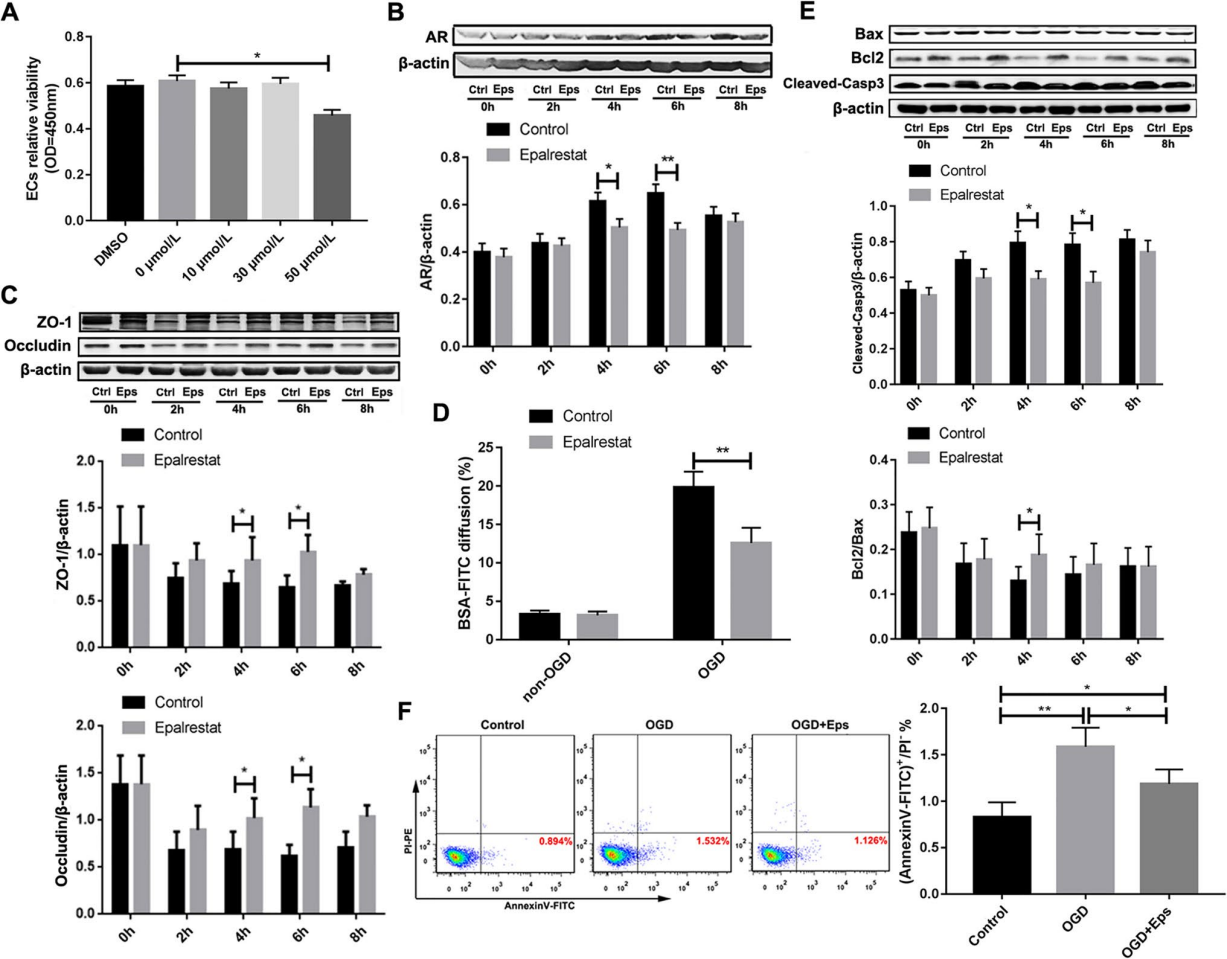
Epalrestat maintains the barrier function of the blood–brain barrier (BBB) by up-regulating tight junction (TJ) proteins and inhibiting endothelial cell (EC) apoptosis after oxygen–glucose deprivation (OGD) *in vitro*. (**A**) Survival of bEnd.3 cells treated with different concentrations of epalrestat (Eps) as assessed by a CCK-8 assay. (*P < 0.05; *n* = 5 per group). (**B**) The expression of aldose reductase (AR) in bEnd.3 cells after OGD treatment at various times as detected by Western blotting (*P < 0.05 and ** *P* < 0.01; *n* = 5 per group). (**C**) The expression of TJ proteins in bEnd.3 cells after OGD treatment at various times as detected by Western blotting. (*P < 0.05; *n* = 5 per group). (**D**) Bovine serum albumin-fluorescein isothiocyanate (BSA-FITC) diffusion rate of confluent bEnd.3 cells grown on 24-well cell culture inserts with indicated concentrations of epalrestat for 1 day with or without OGD exposure for 4 h. (**P < 0.01; *n* = 5 per group). (**E**) The ratio of Bcl2/Bax and the expression of cleaved-caspase 3 in different groups after OGD as detected by Western blotting (*P < 0.05; *n* = 5 per group). (F) The apoptosis of endothelial cells in different groups detected by flow cytometry after OGD. (*P < 0.05 and ** *P* < 0.01; *n* = 5 per group). Data represent the mean ± SD

We stimulated the mouse BMVEC cell line bEnd.3 with different concentrations of epalrestat for 2 days. The CCK-8 assay showed that a high concentration of epalrestat (50 μM) was toxic to the cells. Therefore, we chose 30 μM treatment as the optimal stimulatory concentration of epalrestat *in vitro* for subsequent experiments (Fig. 4A). Western blotting showed that the expression level of AR in endothelial cells increased gradually with the extension of OGD exposure, whereas epalrestat significantly reduced the production of AR at 4 h and 6 h of OGD (Fig. 4B). Moreover, OGD caused extensive loss of TJ proteins compared with that of the control group, whereas epalrestat treatment significantly up-regulated the expression of ZO-1 and occludin in the cells at 4 h and 6 h of OGD exposure (Fig. 4C).

To establish an *in vitro* BBB model, the bEnd.3 cells were cultured on the top of cell culture in inserts for 4 h in OGD or non-OGD conditions 2 d afterepalrestat or no treatment (control). The barrier integrity of the bEnd.3 cell monolayer was assessed using the BSA-FITC transfer rate [47]. As shown in Fig. 4D, the BSA-FITC diffusion rate was suppressed by treatment with 30 μM epalrestat under OGD incubation compared with that of the cells treated without epalrestat after 4 h of OGD exposure.

Western blotting results further showed that the expression level of cleaved-caspase 3 in the endothelial cells increased gradually with the extension of OGD time, whereas epalrestat significantly reduced the production of cleaved-caspase 3 at 4 h and 6 h of OGD exposure. Similarly, the Bcl2/Bax ratio was significantly reduced in the OGD groups compared with that of the non-OGD groups, and this effect was significantly reversed in the epalrestat treatment groups at 4 h of OGD exposure (Fig. 4E).

These results suggested that epalrestat could maintain the barrier function of the BBB after ischemia by reducing the degradation of TJ proteins and inhibiting the apoptosis of endothelial cells.

### Epalrestat Suppresses the Autophagy of BMVECs after Cerebral Ischemia *In Vivo *and *In Vitro*

**Fig. 5:**
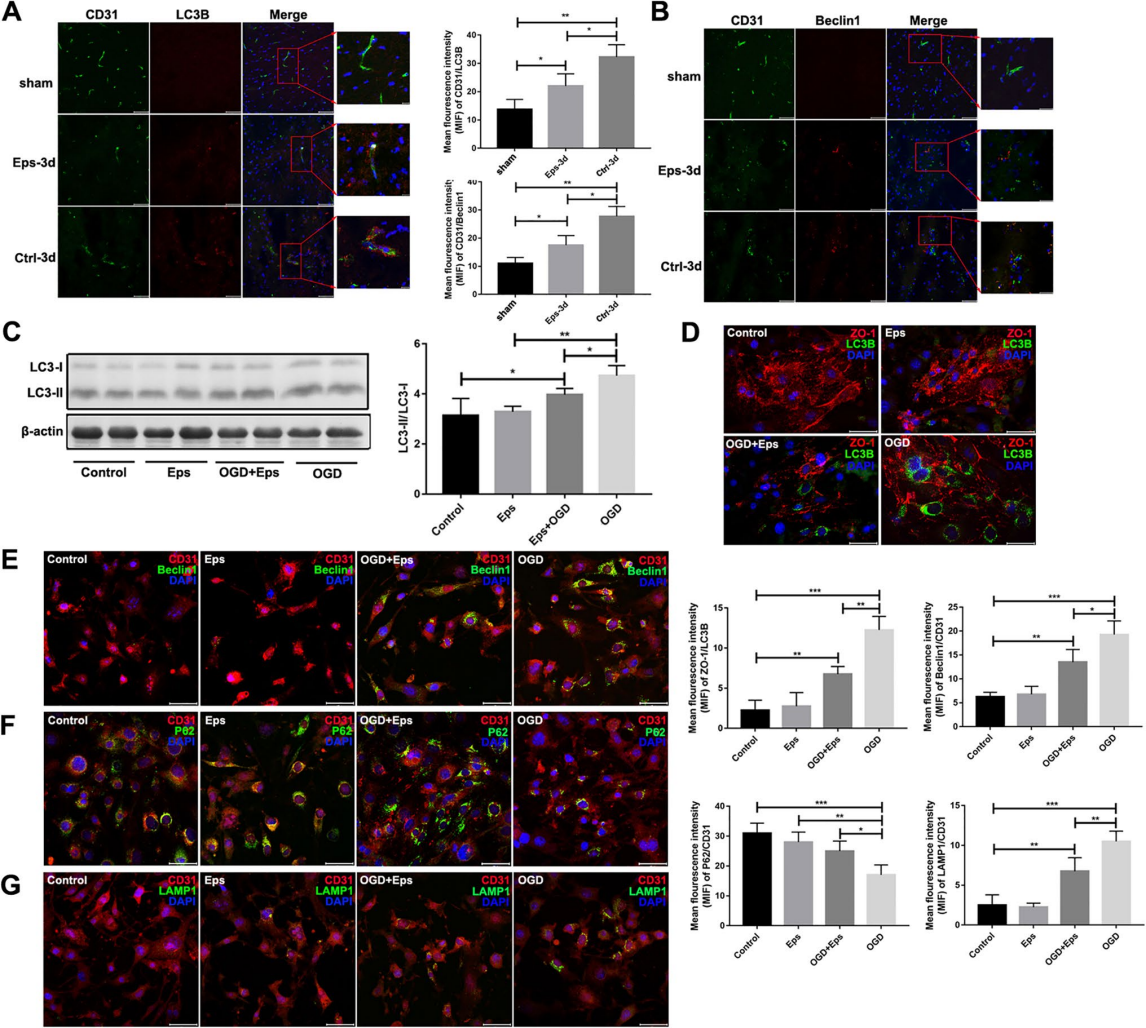
Epalrestat (Eps) suppresses the autophagy of brain microvascular endothelial cells after cerebral ischemia *in vivo* and *in vitro*. (**A**, **B**) Autophagy-related proteins (LC3B and Beclin1, red) and CD31 (green) co-stained around the peri-infarction area in different groups of mice. Scale bar = 10 μm and 20 μm. (**P < 0.05, *** *P* < 0.01;* n* = 5 per group). (**C**) The ratio of LC3-II /LC3-I in bEnd.3 cells with and without oxygen–glucose deprivation (OGD). (**P < 0.05, *** *P* < 0.01; *n* = 5 per group). (**D**) Immunofluorescence double staining of LC3B (green) and ZO-1 (red) expression in bEnd.3 cells after OGD. Scale bar = 20 μm. (**P < 0.01, *** *P* < 0.001; *n* = 5 per group). (E–G) Autophagy-related proteins (Beclin1, P62, and LAMP1) and CD31 co-stained on bEnd.3 cells after OGD. (*P < 0.05, ** *P* < 0.01, *** *P* < 0.001;* n* = 5 per group). Data represent the mean ± SD

Recent reports have shown that AR deficiency can effectively suppress excessive autophagy after cellular injury [48–51]. Consistently, immunofluorescence showed that the expression of the autophagy markers LC3B and Beclin1 in endothelial cells in the ischemic penumbra of the ischemic hemisphere was significantly lower in the epalrestat group than that in the control group *in vivo* 3 days after pMCAL (Fig. 5A-B). Similarly, *in vitro*, the ratio of LC3-II/LC3-I in bEnd.3 endothelial cells in the epalrestat + OGD group was significantly lower than that in the OGD-alone group or in the epalrestat-alone group (Fig. 5C). The mean fluorescent intensity of ZO-1 and LC3B in the epalrestat + OGD group was also lower than that in the OGD-only group (Fig. 5D). Double immunofluorescence staining showed that epalrestat + OGD caused a reduction in the levels of the autophagy-related proteins Beclin1 and LAMP1 compared with those of the OGD-alone group (Fig. 5E, G); however, epalrestat had the opposite effect on P62 (Fig. 5F). These results indicated that epalrestat could inhibit autophagy in BMVECs in an ischemic environment.

### Epalrestat Suppresses OGD-Induced Apoptosis and Autophagy via the AR/AKT/mTOR Signaling Pathway in Endothelial Cells

**Fig. 6:**
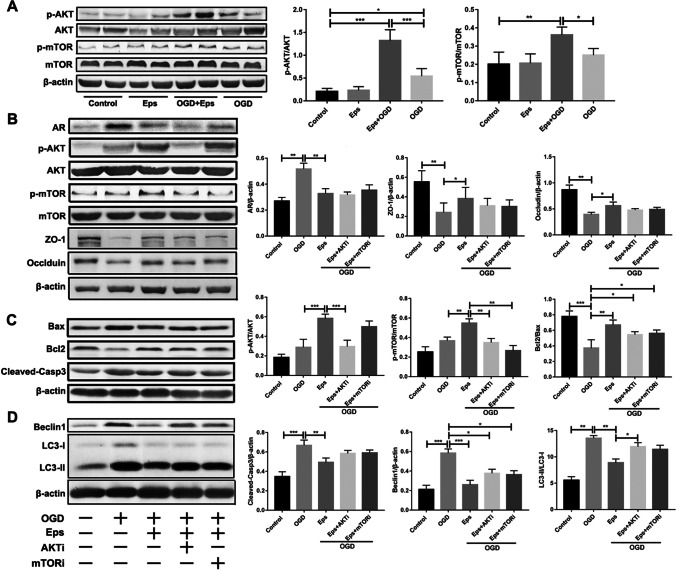
Epalrestat (Eps) suppresses oxygen–glucose deprivation (OGD)-induced apoptosis and autophagy via the AR/AKT/mTOR signaling pathway in endothelial cells. (**A**) Expression of p-AKT, AKT, p-mTOR, and mTOR proteins in different groups after OGD. (*P < 0.05, ** *P* < 0.01, *** *P* < 0.001;* n* = 4 per group). (**B**) Effect of AKT inhibition (AKTi) and mTOR inhibition (mTORi) on aldose reductase (AR), tight junction (TJ) proteins (ZO-1 and occludin), p-mTOR, and p-AKT detected by Western blotting with and without epalrestat under the OGD condition. (*P < 0.05, ** *P* < 0.01, *** *P* < 0.001; *n* = 4 per group). (**C**) Effect of AKTi and mTORi on Bcl2/Bax and cleaved-caspase 3 detected by Western blotting with and without epalrestat under the OGD condition. (*P < 0.05, ** *P* < 0.01, *** *P* < 0.001; *n* = 4 per group). (**D**) Effects of AKTi and mTORi on LC3-II/LC3-I and Beclin1 detected by Western blotting with and without epalrestat under the OGD condition. (*P < 0.05, ** *P* < 0.01, *** *P* < 0.001; *n* = 4 per group). Data represent the mean ± SD

Previous studies have confirmed that excessive AR promotes apoptosis by inactivating the mTOR signaling pathway, whereas PI3K-AKT signaling negatively regulates autophagy induction by activating mTOR, suggesting that inhibition of AKT could promote autophagy by preventing mTOR activation [52]. Therefore, we hypothesized that epalrestat attenuates apoptosis and autophagy by promoting AKT, resulting in mTOR activation. Western blot analysis showed that epalrestat did not affect the expression of p-AKT and p-mTOR under non-OGD conditions. However, co-treatment of epalrestat and OGD markedly increased p-AKT and p-mTOR protein levels compared with those in the OGD-alone group (Fig. 6A). To determine whether AR/AKT/mTOR regulates the expression of TJ proteins, epalrestat, rapamycin (an mTOR inhibitor), and bicalutamide (an AKT inhibitor) were added to bEnd.3 cells *in vitro*. Using different concentrations of inhibitors in cell culture and CCK-8 detection, the optimal concentration of rapamycin and bicalutamide was determined to be 0.5 μm/L and 1 μm/L, respectively (Fig. S4). Western blotting showed that the expression level of AR protein was significantly increased in the OGD-alone group compared with those of the epalrestat + OGD, epalrestat + OGD + bicalutamide, and epalrestate + OGD + rapamycin groups. Moreover, the expression levels of ZO-1 and occludin were significantly increased in the epalrestat + OGD group compared with those in the OGD-alone group. However, the expression of ZO-1 and occludin was down-regulated by the ATK and mTOR inhibitors under the epalrestat + OGD condition. The expression of p-AKT was significantly increased in the epalrestat + OGD group compared with that in the OGD-alone and epalrestat + OGD + bicalutamide groups. Interestingly, the expression of p-mTOR was significantly increased only in the epalrestat + OGD group. These results indicated that epalrestat can up-regulate the expression of TJ proteins in endothelial cells by promoting the AR/AKT/mTOR signaling pathway.

Moreover, Western blotting showed significantly increased expression levels of cleaved-caspase 3 in the OGD-alone group compared with those of the epalrestat + OGD group and control groups, whereas the ratio of Bcl2/Bax was decreased significantly in the OGD group compared with that of the other four groups (Fig. 6C). The ratio of LC3-II/LC3-I in the epalrestat + OGD group was significantly decreased compared with that of the OGD-alone group and the epalrestat + OGD + bicalutamide group, whereas the expression of Beclin1 in the epalrestat + OGD group was significantly decreased compared with that of the OGD-alone group (Fig. 6D). These results suggested that epalrestat may inhibit endothelial cell apoptosis and autophagy under OGD by activating the AR/AKT/mTOR signaling pathway.

## Discussion

**Fig. 7:**
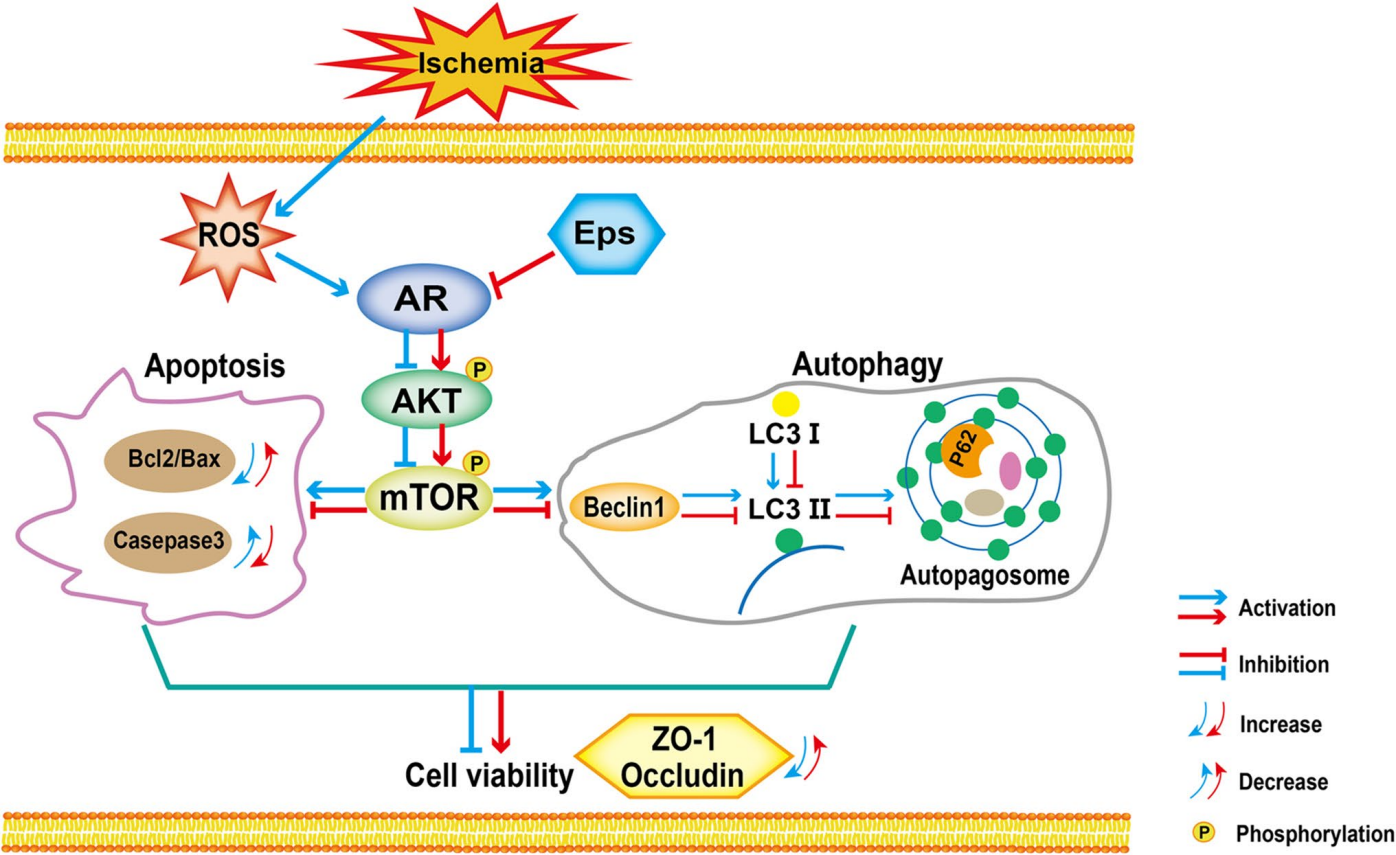
Schematic diagram showing the proposed signaling pathways leading to inhibit apoptosis and autophagy of BMVECs in the cerebral ischemia model after Epalrestat treatment. Epalrestat inhibits BMVECs apoptosis and autophagy, which may promote the activation of AKT / mTOR signaling pathway by inhibiting the activation of AR

This study demonstrates that the AR inhibitor epalrestat has a BBB-protecting function, epalrestat up regulates mTOR phosphorylation through AR/AKT/mTOR signaling pathway, inhibits apoptosis and autophagy of endothelial cells, and maintains the expression level of TJ protein in endothelial cells after cerebral ischemia to protects BBB (Fig. 7).

Previous studies have shown that excessive intracellular production of AR is a key risk factor for aggravating stroke. Yeung et al. [10] indicated that increased expression of AR can aggravate cerebral and retinal ischemia–reperfusion injury in diabetic patients. Lo et al. [53] found that activation of AR can promote iron- and transferrin-related oxidative stress to aggravate cerebral ischemic injury. However, few experimental studies have described the effect of AR on BBB damage in cells and animal models of cerebral ischemia. Our study showed that the expression of AR protein in injured BMVECs after cerebral ischemia was significantly increased, and the use of epalrestat could reduce the expression of AR and effectively improve the function of BMVECs, indicating that inhibition of overproduced AR may be a key strategy to alleviate BBB injury after cerebral ischemia.

Recent experimental studies have demonstrated that the deleterious effects of AR after CNS disease-induced damage involve neuronal dysfunction [54–56] and microglial over activation [29]. Recent studies have also confirmed that the use of AR inhibitors can effectively correct cellular dysfunction and inflammatory responses caused by AR activation. For instance, Iyer et al. [22] demonstrated that epalrestat is the first small-molecule activator of PMM2 enzyme activity with the potential to treat peripheral neuropathy. Song et al. [57] showed that two major AR inhibitors, sorbinil and zopolresta, significantly inhibited the production of inflammatory cytokines (tumor necrosis factor-alpha, interleukin-1β, and interleukin-6) from microglia in response to Aβ stimulation. However, there is no literature on the role of epalrestat in the repair of cerebral ischemic injury. Our study confirmed for the first time that epalrestat can reduce the number of inflammatory infiltrating cells in ischemic regions and attenuate the damage of inflammatory response to the BBB to maintain the stability of the BBB. However, it is still unclear how AR inhibitors improve endothelial cell function after cerebral ischemia. In this study, we found that epalrestat can promote the expression of ZO-1 and occludin in endothelial cells after ischemia both *in vivo* and *in vitro*. Thus, the beneficial influence of epalrestat may involve in the positive regulation of endothelial cell viability.

Epalrestat, as the only AR inhibitor currently approved for clinical application, is used for the treatment of diabetic neuropathy by improving cell activity, and can be more specific and stable targeted inhibition of AR, with lasting efficacy and low side effects [25, 58]. Meantime, epalrestat has stronger brain permeability and can fully enter the central nervous system to play its role [22, 23, 59]. Senthilkumari et al. [60] showed that epalrestat can inhibit apoptosis and appeared to be beneficial in reducing diabetes-related complications in retinal pigment epithelial cells under high-glucose conditions. Wang et al. [16] reported that epalrestat inhibited the activity of AR and reduced the level of high glucose-induced cardiomyocyte apoptosis. In the present study, we also found that epalrestat inhibited BMVEC apoptosis in OGD-injured cells by upregulating the ratio of Bcl2/Bax and down-regulating the level of cleaved-caspase 3, which corresponds with the findings of previous studies. In addition to inhibiting apoptosis, autophagy can be appropriately activated to provide nutrients and energy to the cells under stress conditions such as starvation, hypoxia, and nutrient deficiencies [61]. Geng et al. [40] reported that epalrestat reduced autophagy in hepatocellular carcinoma cells by promoting the mTOR signaling pathway. However, the present study was the first to demonstrate that eparalstat also suppresses excessive autophagy in BMVECs after cerebral ischemia to improve cell viability. These findings suggest that epalrestat can improve the function of endothelial cells, which may be due to the dual effects of inhibiting autophagy and apoptosis after cerebral ischemia. At the same time, both *in vitro* and *in vivo* studies have demonstrated that epalrestat can also promote the expression of tight junction proteins in endothelial cells, enhance tight junctions, and further maintain the stability of the blood–brain barrier.

To date, studies have found that targeted inhibition or activation of the mTOR signaling pathway is an effective way to regulate neuronal apoptosis in the process of cerebral ischemia. For instance, resveratrol has a neuroprotective effect against cerebral ischemia/reperfusion injury by up-regulating the expression of p-JAK2, p-STAT3, p-AKT, p-mTOR, and Bcl2, and down-regulating the expression of cleaved caspase-3 and Bax [62]. In addition, there is accumulating evidence that mTOR kinase responds to stroke to regulate autophagy. Wei et al. [63] indicated that cPKCγ-modulated neuron-specific autophagy improved the neurological outcome of mice following ischemic stroke through the AKT-mTOR pathway. Tang et al. [64] found that exogenous Netrin-1 alleviates damage to ischemic brain tissues and enhances the viability of hypoxic neurons by inhibiting autophagy via the PI3K/mTOR pathway. Consistent with the findings of these previous studies, we found that epalrestat mediates the mTOR pathway in BMVECs after cerebral ischemia and inhibits autophagy and apoptosis. Previous studies have shown that activating the AKT/mTOR signaling pathway can effectively inhibit neuronal apoptosis and autophagy; Therefore, this study is the first to propose and demonstrate that epalrestat can affect endothelial cell viability by modulating the AKT/mTOR pathway, thereby inhibiting apoptosis and excessive autophagy in BMVECs after OGD.

Taken together, our results demonstrate that epalrestat inhibits endothelial cell apoptosis and autophagy by inhibiting AR to upregulate the AKT/mTOR pathway, thereby improving cell activity and maintaining the integrity of the BBB structure. This study provides new mechanistic insights into how epalrestat exerts a neuroprotective effect and attenuates the pathogenesis of stroke, implicating the AR/AKT/mTOR pathway as a potential therapeutic target for stroke patients.

However, many challenges remain in our study. On the one hand, we used epalrestat at a higher concentration in animal experiments than in clinical practice. Although we also observed a therapeutic effect on cerebral ischemia injury, we have not yet had a good method to further detect the specific drug concentration parameters of Epalrestat in ischemic brain tissue. On the other hand, our research is mainly basic experimental work and lacks the presentation of relevant clinical data. Therefore, in future studies, we will focus on the therapeutic effect of epalrestat on clinical ischemic stroke patients.

## Limitation

In this study, we chose three concentrations of epalrestat for in vivo experiments. Although 50 mg/kg is a more suitable concentration compared with 100 mg/kg, whether higher concentrations can have better protective effects needs to be further verified. Meantime, although the side effects of epalrestat in clinical application are very small, which are mainly manifested in the increase of liver enzyme level and gastrointestinal related events such as nausea and vomiting [25, 58], if it is applied to the clinical treatment of cerebral ischemia, it should be further studied whether there are other side effects in the central nervous system. Finally, this study confirmed that epalrestat can regulate apoptosis and autophagy of brain microvascular endothelial cells after cerebral ischemia through AR/AKT/mTOR signal pathway. In order to further study the unique mechanism of epalrestat in treating cerebral ischemic injury, we will use new techniques, such as proteomics and transcriptomics, to find novel and innovative targets.

## Supplementary Information

Below is the link to the electronic supplementary material.

Supplementary file1 (DOCX 470 KB)

## Data Availability

The datasets used and/or analysis during the current study are availability from the corresponding author on reasonable request.

## Abbreviations

AKT: Protein kinase B
AMPK: Adenosine monophosphate activated protein kinase
AR: Aldose reductase; BBB, blood–brain barrier
BMVEC: Brain microvascular endothelial cell
BSA: Bovine serum albumin
CCK-8: Cell Counting Kit-8
CNS: Central nervous system
DAPI: 4′,6-Diamidino-2-phenylindole dihydrochloride
DMEM: Dulbecco's modified Eagle medium
FBS: Fetal bovine serum
FITC: Fluorescein isothiocyanate
mTOR: Mammalian target of rapamycin
OGD: Oxygen-glucose deprivation
PBS: Phosphate-buffered saline
PI3: Phosphoinositide-3 kinase
pMCAL: Permanent middle cerebral artery ligation
ROS: Reactive oxygen species
TJ: Tight junction
TTC: 2,3,5-Triphenyltetrazolium chloride
