# Supplementary material for: The Aldose Reductase Inhibitor Epalrestat Maintains Blood–Brain Barrier Integrity by Enhancing Endothelial Cell Function during Cerebral Ischemia
Source: Mol Neurobiol. 2023 Mar 20;60(7):3741–57. doi: 10.1007/s12035-023-03304-z (PMC10224835; doi:10.1007/s12035-023-03304-z)
Supplement: Supplementary file 1 — Supplementary file1 (DOCX 470 KB) [file 12035_2023_3304_MOESM1_ESM.docx]

**The aldose reductase inhibitor epalrestat maintains blood-brain barrier integrity by** **enhancing endothelial cell function during cerebral ischemia**

Tongshuai Zhang^a,1^, Jinrong Wu^b,1^, Xinmin Yao^c,1^, Yao Zhang^a^, Yue Wang^d^, Yang Han^a^, Yun Wu^e^, Zhenyu Xu^a^, Jing Lan^a^, Siyu Han^a^, Haifeng Zou^a^, Qixu Sun^f^, Dandan Wang^g*^, Jingyu Zhang^h*^, Guangyou Wang^a*^

^a^Department of Neurobiology, Heilongjiang Provincial Key Laboratory of Neurobiology, Harbin Medical University, Harbin, Heilongjiang, 150081, China

^b^Department of Anaesthesiology, The First Affiliated Hospital of Harbin Medical University, Harbin, Heilongjiang, 150001, China

^c^Traditional Chinese medicine,Heilongjiang University of Chinese Medicine, Harbin, Heilongjiang, 150040, China

^d^Department of Anesthesiology,Second Affiliated Hospital of Air Force Medical University, Xi'an, Shaanxi, 710032, China

^e^The Medical Department of Neurology, The Second Affiliated Hospital of Harbin Medical University, Harbin, Heilongjiang, 150001, China

^f^Department of Gastroenterology, Penglai people's Hospital, Yantai, Shandong, [264117](https://www.youbianku.com/264117), China.

^g^Wu Lian De Memorial Hospital, The First Affiliated Hospital of Harbin Medical University, Harbin, Heilongjiang, 150001, China

^h^The Medical Department of Neurology, The Fourth Affiliated Hospital of Harbin Medical University, Harbin, Heilongjiang, 150001, China

*Corresponding authors:

Guangyou Wang, Department of Neurobiology, Harbin Medical University, Harbin, Heilongjiang, 150081, China

Jingyu Zhang, The Medical Department of Neurology, The Fourth Affiliated Hospital of Harbin Medical University, Harbin, Heilongjiang, 150001, China

Dandan Wang, Wu Lian De Memorial Hospital, The First Affiliated Hospital of Harbin Medical University, Harbin, Heilongjiang, 150001, China

Tel.: +86 451 86662943; Fax: +86 451 87502363.

E-mail address: wangguangyou@hrbmu.edu.cn (Guangyou Wang).

zhangjingyuyx@163.com (Jingyu Zhang)

wangdandan1973@126.com (Dandan Wang)

^1^These authors contributed equally to this work.

**Supplementary material**

**
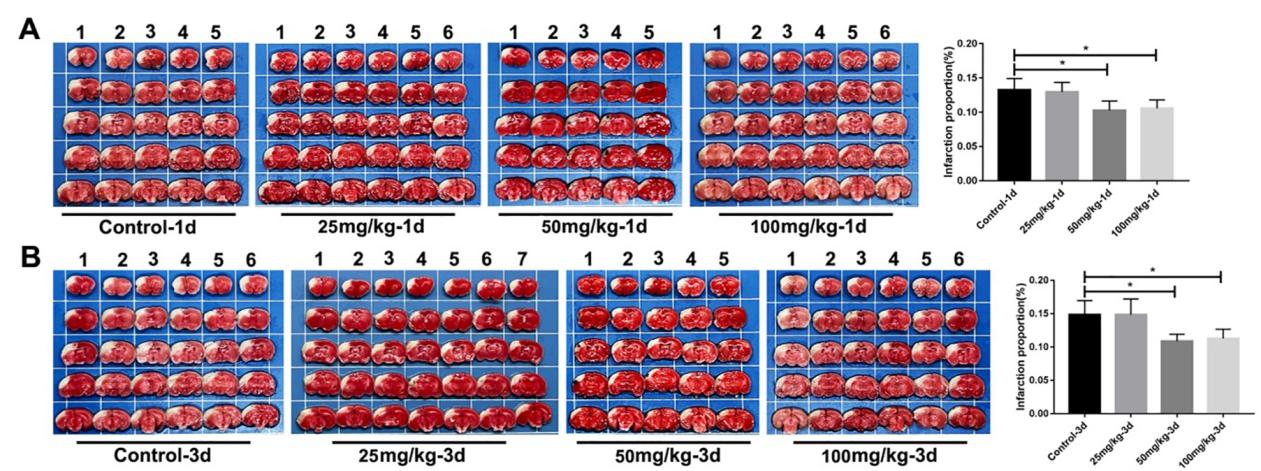
**

**Supplementary Figure S1. Effect of different concentrations of epalrestat on ischemic brain volume** (A) One day and (B) three days after cerebral ischemia, mice were given different concentrations of epalrestat by gavage, the cerebral ischemic area was detected by TTC staining. **P* < 0.05; *n* = 5 per group. The data represent the mean ± SD.


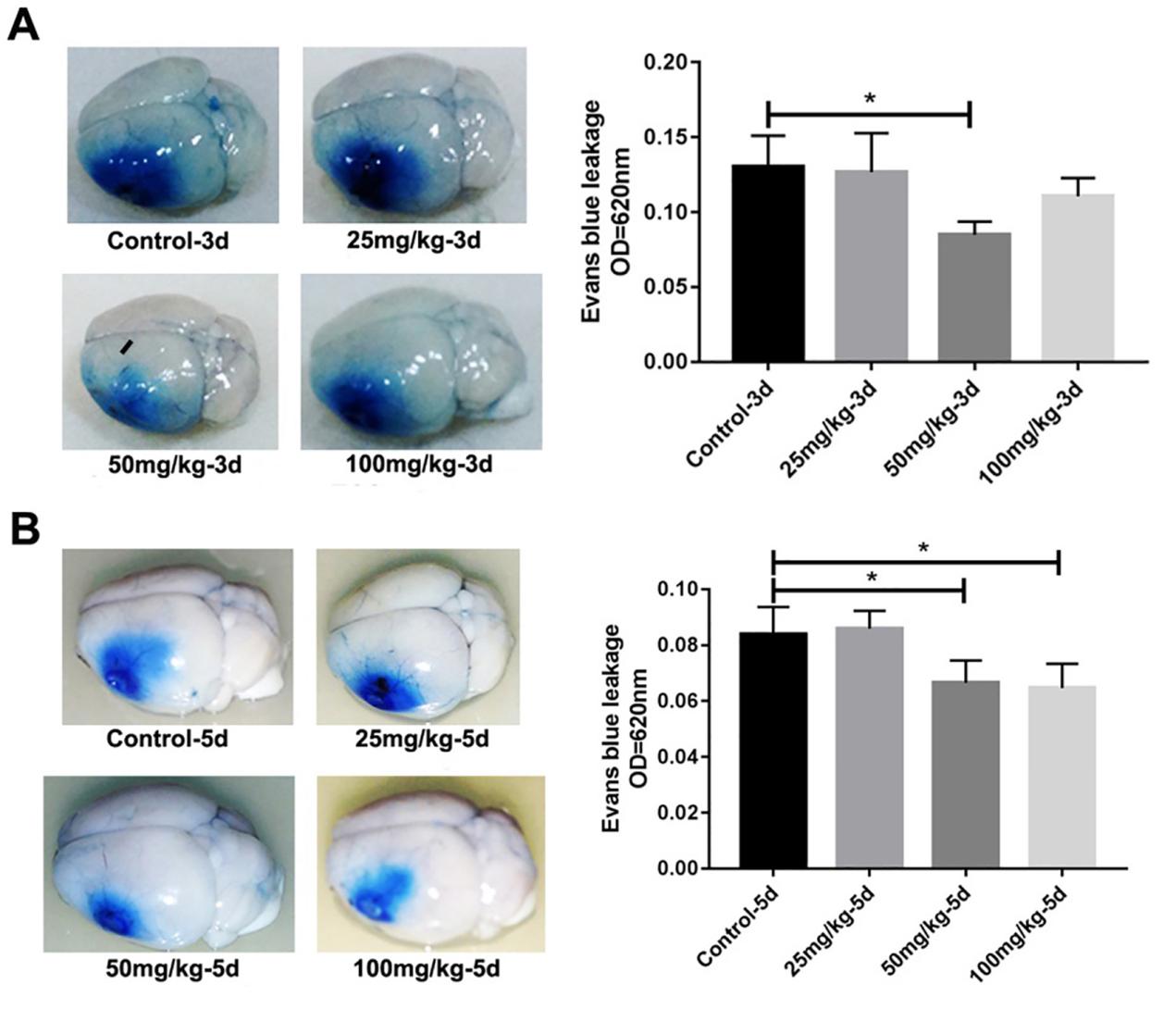


**Supplementary Figure S2. Effect of different concentrations of epalrestat on blood brain barrier function after cerebral ischemia** (A) Three days and (B) Five days after cerebral ischemia, mice were given different concentrations of epalrestat by gavage, the blood-brain barrier permeability was detected by EB staining. **P* < 0.05; *n* = 5 per group. The data represent the mean ± SD.


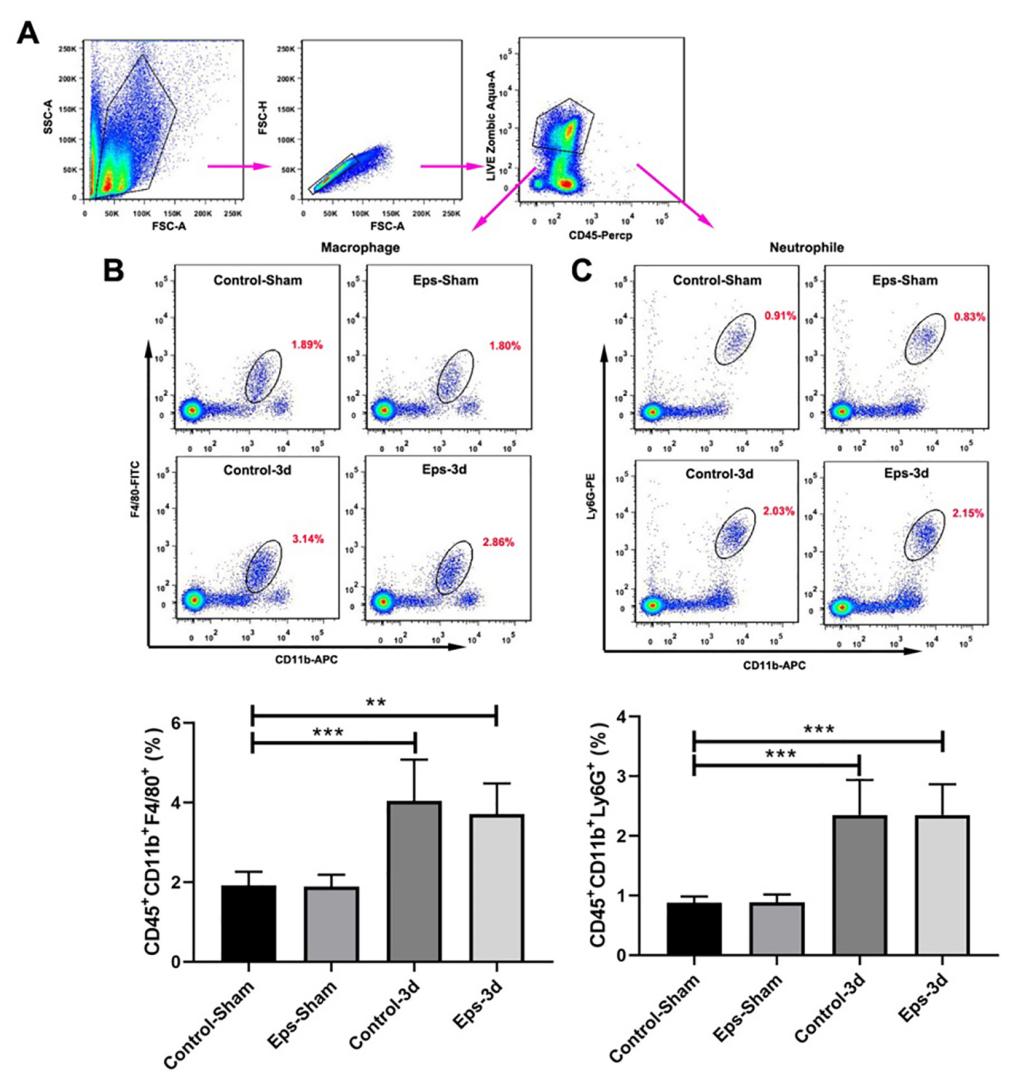


**Supplementary Figure S3. Effect of epalrestat on macrophages and neutrophils in peripheral blood circulation after cerebral ischemia** (A) The location of the active leukocyte mass in the peripheral blood was determined using the first gate Fixable Viability Dye eFluor™ 780 (FVD) and CD45-Percp. (B) Macrophages in the blood were labeled with CD45^+^CD11b^+^F4/80^+^,and (C) neutrophils were labeled with CD45^+^CD11b^+^Ly6G^+^. ***P* < 0.01, ****P* < 0.001; *n* = 5 per group. The data represent the mean ± SD.


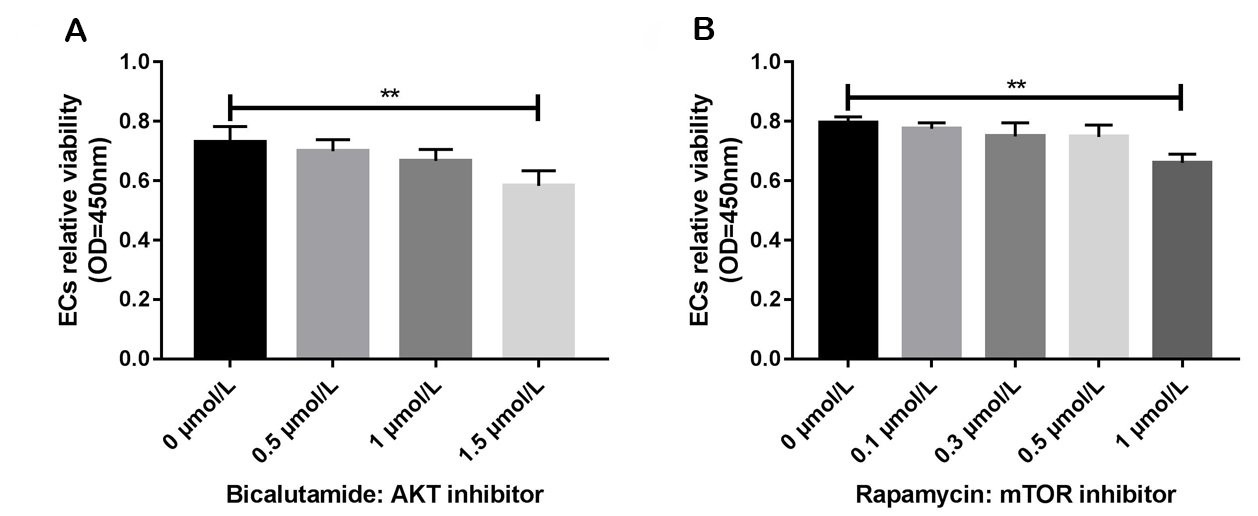


**Supplementary Figure S4. Cytotoxicity analysis of various concentrations of the inhibitors** (A) bicalutamide and (B) rapamycin using the CCK-8 assay in bEnd.3 mouse endothelial cells (ECs). ***P* < 0.01; *n* = 5 per group. The data represent the mean ± SD.
